# Depression Significantly Predicts Clinical Functioning Among Hispanic older adults

**DOI:** 10.1002/alz.088084

**Published:** 2025-01-03

**Authors:** Andres Duarte, Dilianna Padron, Patricia Garcia, Lisandra Mendoza, Jacob Fiala, Michael Marsiske, Ranjan Duara, Miriam J. Rodriguez

**Affiliations:** ^1^ Albizu University, Doral, FL USA; ^2^ Rehabilitation Hospital of Indiana, Indianapolis, IN USA; ^3^ Bay Pines VA Healthcare System, Bay Pines, FL USA; ^4^ Florida State University College of Medicine, Miami, FL USA; ^5^ University of Florida, Gainesville, FL USA; ^6^ Florida Alzheimer’s Disease Research Center, Gainesville, FL USA; ^7^ 1Florida Alzheimer’s Disease Research Center, Miami, FL USA; ^8^ Indiana University, Bloomington, IN USA

## Abstract

**Background:**

Hispanics are disproportionately affected by Alzheimer’s disease and related dementias (ADRD) when compared to non‐Hispanic Whites (NHW). Previous studies have shown that neuropsychiatric symptoms can further exacerbate clinical functioning among ADRD patients, leading to increase in caregiver burden and poorer quality of life. However, most studies in this area have focused on predominately Caucasian samples, and relationships between emotional and clinical functioning among Hispanic older adults at risk of ADRD are poorly understood. The aim of the current study was to examine predictive associations between emotional and clinical functioning among Hispanic ADRD older adults at risk of developing ADRD.

**Methods:**

The Geriatric Depression Scale (GDS) and the modified Clinical Dementia Rating scale (mCDR) were administered to Hispanic (n = 159) and NHW (n = 101) participants from the 1Florida Alzheimer’s Disease Research Center (ADRC). Participants were aged 60‐90 years (Hispanics: M = 70.87, SD = 7.28; NHW: M = 73.24, SD = 7.72) and were either cognitive normal (n = 52) or diagnosed with Mild Cognitive Impairment (MCI, n = 208). Multiple linear regression analyses controlling for age and diagnosis were applied to examine predictive relationships between the emotional and functional measures for the two ethnic groups separately.

**Results:**

Results showed significant positive relationship between the mCDR and the GDS (β = 0.01, SE = 0.00, t = 2.33, p > .05) among the Hispanic sample and interaction effects between diagnosis and GDS scores were not significant (β = 0.00, SE = 0.01, t = ‐0.41, p = 0.68). No significant associations were noted among the NHW group (β = 0.01, SE = 0.00, t = 1.13, p = 0.26).

**Conclusion:**

Depression significantly predicted poorer clinical functioning among the Hispanic group only even when controlling for the effects of diagnosis. These findings indicate that the effect of emotional symptoms has a clinical impact on functioning among Hispanic older adults and may be related to increased risk of further decline and ADRD diagnosis. These results have important implications for dementia care. Future studies should examine these associations as they relate to ADRD risk and longitudinal clinical functioning.

